# Association between renal damage markers and carotid atherosclerosis in Afro-descendants with hypertension belonging to a minority ethnic group from Brazil

**DOI:** 10.1080/0886022X.2018.1496932

**Published:** 2018-10-03

**Authors:** Dyego José de Araújo Brito, Elisangela Milhomem dos Santos, Raimunda Sheyla Carneiro Dias, Isabela Leal Calado, Gyl Eanes Barros Silva, Joyce Santos Lages, Francisco das Chagas Monteiro Júnior, Alcione Miranda dos Santos, Natalino Salgado Filho

**Affiliations:** a Postgraduate Program of Health Science, Federal University of Maranhão, São Luís, Brazil;; b Nephrology Division, University Hospital of Federal University of Maranhão, São Luís, Brazil;; c Department of Nursing, Federal University of Maranhão, São Luís, Brazil;; d Department of Nutrition, Federal University of Maranhão, São Luís, Brazil;; e Department of Pathology, Federal University of Maranhão, São Luís, Brazil;; f Department of Public Health, Federal University of Maranhão, São Luís, Brazil;; g Cardiology Division, University Hospital of Federal University of Maranhão, São Luís, Brazil;; h Department of Medicine I, Federal University of Maranhão, São Luís, Brazil

**Keywords:** Renal damage markers, carotid atherosclerosis, hypertension, minority ethnic group, Afro-descendants

## Abstract

Ethnicity appears to play an important role in the prevalence and severity of hypertension, renal disease, and atherosclerosis. A cross-sectional study was conducted, including 206 Afro-descendants with hypertension, living in the remaining quilombo communities. These subjects underwent a carotid intima-media thickness (CIMT) assessment. The presence of renal injury was assessed by: (1) The glomerular filtration rate (GFR) estimated by the formula CKD-EPI using creatinine and cystatin C and (2) Albuminuria (ACR ≥30 mg/g). The Poisson distribution model was set with robust variance to identify factors associated with carotid atherosclerosis. The statistical analysis was performed using the Stata 12.0 software, adopting a significance level of 5%. Most subjects were women (61.65%); the average age was 61.32 (±12.44) years. Subjects (12.62%) were identified with GFR <60 mL/min/1.73 m^2^ and 22.8% with albuminuria. Patients (59.22%) presented with a high CIMT. In the adjusted regression model, age ≥60 years (PR: 1.232 [CI 95%:1.091–1.390], *p* value = .001), ACR ≥30 mg/g (PR: 1.176 [CI 95%: 1.007–1.373], *p* = .040), and GFR/CKD-EPI using cystatin C (PR: 1.250 [CI 95%: 1.004–1.557], *p* = .045) were independently associated with carotid atherosclerosis. The occurrence of atherosclerotic lesions was high in the studied group. Age, albuminuria, and GFR (estimated by the formula CKD-EPI using cystatin C) influenced the prevalence of carotid atherosclerosis.

## Introduction

Cardiovascular diseases have been historically considered as diseases of the developed countries. However, these disorders are rapidly contributing to an increased morbidity in the developing regions with varied racial/ethnic groups [1,2].

Ethnicity appears to play an important role in the prevalence and severity of systemic arterial hypertension (SAH), atherosclerosis, and renal disease. The Black race is a strong predisposing factor to SAH, leaving the Afro-descendant population exposed to the risk of a more severe hypertension, as well as a greater risk for angina, heart attack, and sudden death [3].

The assessment of carotid atherosclerotic disease has revealed an association with ethnicity in several studies [4–6]. Williams et al. [7] studied the relationship between aggression and subclinical carotid atherosclerosis in 14,098 subjects of the *Atherosclerosis Risk in Communities Study (ARIC)* cohort. They observed that the carotid intima-media thickness (CIMT) was greater in Black men with an aggressive profile, when compared to other sexes and ethnicity groups. In a cardiovascular health study, which evaluated 4926 White Caucasian subjects and 244 Black subjects of both sexes, aged 65 years or over, it was found that the CIMT was significantly greater in the Black population when compared to the White population, even after adjusting for cardiovascular risk factors [8].

The differences in the occurrence of cardiovascular disease between ethnic groups cannot be completely explained by traditional risk factors, despite a higher occurrence of arterial hypertension and diabetes mellitus in the Black population [9]. In conclusion, the association between atherosclerosis and cardiovascular disease (CVD) among the different ethnic groups and the impact of risk factors on this association is not yet well established. Thus, the *American Heart Association* has recommended the investigation of atherosclerotic cardiovascular diseases in all ethnic groups, including short-term and long-term risk analyses to fill this knowledge gap [10].

This study aims to investigate the influence of renal disease markers on the prevalence of carotid atherosclerotic disease among African-American residents in the remaining quilombo communities in the northern state of Maranhão, Brazil.

## Methods

### Study design

This is a cross-sectional analysis including the Afro-descendants with hypertension who participated in the PREVRENAL: ‘*Prevalence of Chronic Renal Disease in the municipality of São Luís and Alcântara Quilombola communities, State of Maranhão*’ study, which evaluated 1539 African-American people from the 32 remaining quilombo communities in northeastern Brazil (municipality of Alcântara, State of Maranhão), between August 2012 and April 2013.

### Study sample

The sample size calculation was estimated considering the population of the Afro-descendants with hypertension previously diagnosed in the PREVRENAL study (*n* = 439), using a two-tailed hypothesis test (test power =80%, confidence level = 95%, prevalence of carotid atherosclerosis in the exposed population = 65% [11], and prevalence ratio = 1.5). Thus, the sample size was estimated as 164 individuals. With the purpose of covering any losses during the data collection process, it was decided to increase the sample by up to 20%, totaling 197 individuals.

The individuals with hypertension included in this study were randomly selected through a list obtained from the PREVRENAL database, totaling 206 patients.

### Data collection

The PREVRENAL pre-codified questionnaire was employed for data collection. It sought information concerning demographic, socioeconomic, behavioral, and clinical data of the patients with hypertension, who were included in this study. All researchers were previously trained to fill out the questionnaires. The interviews were conducted at the selected interviewers’ homes.

### Clinical and laboratory variables

Education was presented in years of study. Age was categorized into age groups. Smoking was defined as current consumption of ≥1 cigarette/d. Weight (kg) and height (m) were measured to calculate the body mass index (BMI, calculated in kg/m^2^). Individuals with BMI >30 kg/m^2^ were classified as obese [12].

Diabetes mellitus (DM), history of stroke (S), acute myocardial infarction (AMI)/angina, and use of medications were referred to by the study participants, based on their history of a previous medical diagnosis.

The BP assessment was performed by the oscillometric method, using an Omron 705-IT device. Three measures of systolic (SBP) and diastolic (DBP) blood pressure were made, in which the average of the last two measurements was used.

Patients who had a previous diagnosis, or blood pressure levels equal to or exceeding 140 mmHg (SBP) and/or 90 mmHg (DBP), in two measurements at different times were considered to have systemic arterial hypertension (during the application of the questionnaire, on the first day; and at the time of the anthropometric measurements and collection of biological material, on the second day).

Venous blood samples were taken after a 12 h fasting, and included the following biochemical dosages: creatinine, cystatin C, blood count, glucose, uric acid, lipid profile, ultra-sensitive c-reactive protein (CRP). The uric acid was considered high, when it was above 7 mg/dL for men and above 6 mg/dL for women.

The dyslipidemia was categorized as (1) isolated hypercholesterolemia: LDL isolated elevation (≥160 mg/dL); (2) isolated hypertriglyceridemia: triglycerides elevation (≥150 mg/dL); (3) mixed hyperlipidemia: LDL ≥160 mg/dL and triglycerides ≥150 mg/dL; and (4) low HDL: <40 mg/dL in men and <50 mg/dL in women [13].

### Renal disease markers

The glomerular filtration rate (GFR) was estimated from the formula derived from the CKD-EPI study, using the value of creatinine [14] and cystatin C [15] as a reference for the calculation. To form the groups, the individuals were characterized as patients with normal or reduced GFR. GFR was considered reduced when <60 mL/min/1.73 m^2^.

The value of urinary albumin was obtained from the albuminuria/creatinine ratio (ACR), of an isolated sample of the second urine of the day. Patients with laboratory urinary value ≥30 mg/g were defined as having albuminuria.

### Assessment of the CIMT

To assess the CIMT, a GE Vingmed Ultrasound device, model Vivid3 (Horten, Norway) was used. The ultrasound examination of the carotid was performed by a single experienced examiner, blinded to the clinical and laboratory data and risk classification of each patient. After a rest of at least 10 min in supine position with the neck in discreet hyperextension, an evaluation of the carotid system was performed bilaterally. The CIMT was measured in the distal wall (the farthest wall from the transducer) of the common carotid, 1 cm proximally to its bifurcation, according to the current recommendations. The measurement consisted of the distance between two echogenic lines represented by the lumen-intima and media-adventitia interfaces of the arterial wall. Values were considered normal when CIMT <0.9 mm and amended when CIMT ≥ 0.9 mm [16].

#### Statistical analysis

First, a descriptive analysis of the study variables was carried out. Numeric variables were presented as mean and standard deviation, and the categorical variables were presented as frequencies and percentages. The association between independent variables and carotid atherosclerosis was examined in the bivariate analysis by the chi-squared test, with a significance level of 5%. The Poisson regression with robust variance was adopted to investigate how the prevalence of carotid atherosclerosis could be influenced by various explanatory variables. Considering this, the multiple Poisson regression was carried out for the adjusted analysis. In the multiple model, the variables with *p* < .20 in the bivariate analysis, and only those with *p* < .05 remained in the model. The data were analyzed using the Stata 12.0 software.

#### Ethical approval

All procedures performed in this study were in accordance with the ethical standards of the institutional and/or national research committee and with the 1964 Helsinki Declaration and its later amendments or comparable ethical standards. The research protocol was approved by the Research Ethics Committee of the University Hospital of Federal University of Maranhão (consolidated opinion No. 41492/2012), in compliance with the requirements of the Resolution of the National Health Council No. 466/12 and its supplementary norms for research involving human beings.

## Results

The study sample consisted of 206 Afro-descendants with hypertension, mostly women (*n* = 127; 61.65%), with a mean age of 61.32 (±12.44) years. Ninety-four (45.63% of the sample) individuals were aged less than 60 years. Twenty-six subjects with diabetes mellitus (12.62% of the sample), 19 subjects with a history of stroke (9.22% of the sample), 46 subjects with obesity (22.33% of the sample), and 58 smoker subjects (28.15% of the sample) were identified. The measurement of systolic blood pressure (SBP) revealed SBP values of ≥140 mmHg in 158 individuals (76.70% of the sample) and diastolic blood pressure (DBP) ≥ 90 mmHg in 92 cases (44.66% of the sample). The distribution of the clinical-epidemiological characteristics, according to the CIMT, are shown in Table 1.

**Table 1.** Distribution of the clinical-epidemiological characteristics in accordance with the carotid intima-media thickness.

Variables	*n* (%)	CIMT < 0.9 mm	CIMT ≥ 0.9 mm	*p* value
		*n* (%)	*n* (%)	
Sex				
Male	79 (38.35)	24 (30.38)	55 (69.62)	.017
Female	127 (61.65)	60 (47.24)	67 (52.76)	
Age				
<60 years	94 (45.63)	57 (60.64)	37 (39.36)	<.001
60–79 years	99 (48.06)	26 (26.26)	73 (73.74)	
≥80 years	13 (6.31)	1 (7.69)	12 (92.31)	
Education				
0–4 years	180 (87.38)	69 (38.33)	111 (61.67)	.158
5–8 years	13 (6.31)	8 (61.54)	5 (38.46)	
>8 years	13 (6.31)	7 (53.85)	6 (46.15)	
History of DM				
No	180 (87.38)	79 (43.89)	101 (56.11)	.017
Yes	26 (12.62)	5 (19.23)	21 (80.77)	
History of Stroke				
No	187 (90.78)	79 (42.25)	108 (57.75)	.178
Yes	19 (9.22)	5 (26.32)	14 (73.68)	
Use of ACEI/ARB				
No	114 (55.34)	52 (45.61)	62 (54.39)	.116
Yes	92 (44.66)	32 (34.78)	60 (65.22)	
Use of Statin				
No	199 (96.60)	82 (41.21)	117 (58.79)	.504
Yes	7 (3.40)	2 (28.57)	5 (71.43)	
Use of ASA				
No	189 (91.75)	81 (42.86)	108 (57.14)	.043
Yes	17 (8.25)	3 (17.65)	14 (82.35)	
Smoking				
No	148 (71.84)	66 (44.59)	82 (55.41)	.075
Yes	58 (28.16)	18 (31.03)	40 (68.97)	
BMI (kg/m²)				
<30	160 (77.67)	61 (38.13)	99 (61.88)	.149
≥30	46 (22.33)	23 (50.00)	23 (50.00)	
PAS (mmHg)				
<140	48 (23.30)	26 (54.17)	22 (45.83)	.031
≥140	158 (76.70)	58 (36.71)	100 (63.29)	
DBP (mmHg)				
<90	114 (55.34)	44 (38.60)	70 (61.40)	.478
≥90	92 (44.66)	40 (43.48)	52 (56.52)	

CIMT: carotid intima-media thickness; DM: diabetes mellitus; S: stroke; AMI: acute myocardial infarction; ACEI/ARB: angiotensin converting enzyme inhibitor/angiotensin-II-receptor blocker AT1; ASA: acetylsalicylic acid; BMI: body mass index; SBP: systolic blood pressure; DBP: diastolic blood pressure.

In 16 cases (7.77% of the sample), the patients had a GFR <60 mL/min/1.73 m^2^ calculated by the formula CKD-EPI using the serum creatinine, and 26 patients (12.62% of the sample) had a reduced GFR assessed by the CKD-EPI formula using cystatin C as a marker. Albuminuria (≥30 mg/g of creatinine) occurred in 47 cases (22.8% of the sample).

CIMT was assessed in 122 patients (59.22% of the sample), and calcified plates were identified in 100 patients (48.54% of the samples). Carotid atherosclerosis was more frequent in male Afro-descendants (69.62% of the sample), those aged between 60 and 79 years (73.74% of the sample), those aged ≥80 years (92.31% of the sample), those with a history of diabetes mellitus (80.77% of the sample), those using acetylsalicylic acid (ASA) (82.35%), those with SBP ≥140 mmHg (63.29%), those with albuminuria/creatinine ratio (ACR) ≥ 30 mg/g (72.34%), those with GFR <60 mL/min/1.73 m^2^ estimated by formula CKD-EPI creatinine (87.50%) and CKD-EPI cystatin C (92.31%), those without isolated hypertriglyceridemia (59.55%), those with mixed hyperlipidemia (75.00%), and those with hyperuricemia (75.86%), as shown in Tables 1 and 2.

**Table 2.** Distribution of the laboratorial characteristics in accordance with the carotid intima-media thickness.

Variables	*n* (%)	CIMT < 0.9 mm	CIMT ≥ 0.9 mm	*p* value
		*n* (%)	*n* (%)	
ACR (mg/g)				
<30	159 (77.18)	71 (44.65)	88 (55.35)	.037
≥30	47 (22.82)	13 (27.66)	34 (72.34)	
CRP (mg/dL)				
<1	186 (90.29)	76 (40.86)	110 (59.14)	.790
1–3	16 (7.77)	7 (43.75)	9 (56.25)	
>3	4 (1.94)	1 (25.00)	3 (75.00)	
GFR/CKD-EPIcr (mL/min/1.73 m²)				
≥60	190 (92.23)	82 (43.16)	108 (56.84)	.017
<60	16 (7.77)	2 (12.5)	14 (87.50)	
GFR/CKD-EPIcy (mL/min/1.73 m²)				
≥60	180 (87.38)	82 (97.62)	98 (80.33)	<.001
<60	26 (12.62)	2 (7.69)	24 (92.31)	
Isolated Hypercholesterolemia				
No	178 (86.41)	72 (40.45)	106 (59.55)	.810
Yes	28 (13.59)	12 (42.86)	16 (57.14)	
Isolated Hypertriglyceridemia				
No	160 (77.67)	59 (36.88)	101 (63.13)	.034
Yes	46 (22.33)	25 (54.35)	21 (45.65)	
Mixed Hyperlipidemia				
No	170 (82.52)	75 (44.12)	95 (55.88)	.034
Yes	36 (17.48)	9 (25.00)	27 (75.00)	
Low HDL				
No	114 (55.34)	41 (35.96)	73 (64.04)	.118
Yes	92 (44.66)	43 (46.74)	49 (53.26)	
Fasting Blood Glucose				
<100	77 (37.38)	50 (38.76)	34 (44.16)	.446
≥100	129 (62.62)	43 (55.84)	79 (61.24)	
Uric Acid				
Normal	177 (85.92)	77 (43.50)	100 (56.50)	.049
High	29 (14.08)	7 (24.14)	22 (75.86)	

CIMT: carotid intima-media thickness; ACR: Albumin/Creatinin Ratio; CPR: ultra-sensitive C-reactive protein; GFR: estimated glomerular filtration rate; CKD-EPIcr: Chronic Kidney Disease Epidemiology Collaboration (use of creatinine); CKD-EPIcy: Chronic Kidney Disease Epidemiology Collaboration (use of cystatin C); HDL: high density lipoprotein.

Tables 3 and 4 present the estimates of the prevalence ratio (PR) in the non-adjusted analysis. It must be pointed out that there was greater prevalence of CIMT among men (PR = 1.32, *p* = .014), in individuals aged between 60 and 79 years (PR = 1.87, *p* < .001) and ≥80 years (PR = 2.35, *p* < .001), those with diabetes mellitus (PR = 1.44, *p* values = .002), those using ASA (PR = 1.84, *p* = .001), those with ACR ≥30 mg/g of creatinine (PR = 1.31, *p* = .020), those with GFR <60 mL/min/1.73 m^2^ by the formula using creatinine (PR = 1.54, *p* values < .001), and using the CKD-EPI cystatin C (PR = 1.70, *p* < .001), those with mixed hyperlipidemia (PR = 1.34, *p* = .013) and hyperuricemia (PR = 1.47, *p* < .001).

**Table 3.** Non-adjusted regression model for evaluation of association of carotid intima-media thickness with clinical and epidemiological variables.

Variables	PR	CI (95%)	*p* value
Sex			
Female	1		
Male	1.32	1.05–1.64	.014
Age			
<60 years	1		
60–79 years	1.87	1.41–2.47	<.001
≥80 years	2.34	1.74–3.15	<.001
Education			
0–4 years	1		
5–8 years	0.62	0.31–1.25	.186
>8 years	0.75	0.41–1.36	.344
History of DM			
No	1		
Yes	1.44	1.14–1.80	.002
History of Stroke			
No	1		
Yes	1.28	0.94–1.71	.107
Use of ACEI/ARB			
No	1		
Yes	1.19	0.95–1.50	.114
Use of Statin			
No	1		
Yes	1.21	0.74–1.97	.430
Use of ASA			
No	1		
Yes	1.44	1.11–1.85	.005
Smoking			
No	1		
Yes	1.24	0.99–1.55	.057
BMI (kg/m²)			
<30	1		.184
≥30	0.81	0.59–1.10	
SBP (mmHg)			
<140	1		
≥140	1.38	0.99–1.92	.056
DBP (mmHg)			
<90	1		.483
≥90	0.92	0.73–1.16	

PR: prevalence ratio; CI: confidence interval; DM: diabetes mellitus; S: stroke; MI: myocardial infarction; ACEI/ARB: angiotensin-converting enzyme inhibitor inhibitor/angiotensin-II (AT1) receptor blocker; ASA: acetylsalicylic acid; BMI: body mass index; SBP: systolic blood pressure; DBP: diastolic blood pressure.

**Table 4.** Model of regression not adjusted to evaluate the association of carotid intima-media thickness with laboratory variables.

Variables	PR	CI (95%)	*p* value
RAC (mg/g)			
<30	1		
≥30	1.31	1.04–1.63	.020
CRP (mg/dL)			
>3	1		
1–3	1,27	0.71–2.26	.422
<1	0.95	0.60–1.49	.827
GFR/CKD-EPIcr (mL/min/1.73 m²)			
<60	1		
≥60	1.54	1.23–1.92	<.001
GFR/CKD-EPIcy (mL/min/1.73 m²)			
<60	1		
≥60	1.70	1.42–2.01	<.001
Isolated Hypercholesterolemia			
No	1		
Yes	0.96	0.68–1.35	.814
Isolated Hypertriglyceridemia			
No	1		
Yes	0.72	0.51–1.01	.060
Mixed Hyperlipidemia			
No	1		
Yes	1.34	1.06–1.69	.013
Low HDL			
No	1		
Yes	0.83	0.65–1.05	.126
Fasting Blood Glucose			
<100	1		
≥100	1.09	0.86–1.39	.455
Uric Acid			
Normal	1		
High	1.34	1.05–1.71	.018

PR: prevalence ratio; CI: confidence interval; RAC: Ratio: Albumin/Creatinin; CPR: high-sensitive C-reactive protein; GFR: glomerular filtration rate; estimated CKD-EPIcr: Chronic Kidney Disease Epidemiology Collaboration (use of creatinine); CKD-EPIcy: Chronic Kidney Disease Epidemiology Collaboration (use of cystatin C); HDL: high density lipoprotein.

In the adjusted analysis, the following remained statistically associated: age ≥60 years (PR = 1.23, *p* values = .001), ACR ≥30 mg/g (PR = 1.18, *p* = .040) and GFR <60 mL/min/1.73 m^2^ assessed by formula using cystatin C only (PR = 1.25, *p* = .045).

## Discussion

This study included the Afro-descendants with hypertension residing in the remaining quilombo communities in northeastern Brazil. It aimed to evaluate the association of renal disease markers with carotid atherosclerosis, and identified a high prevalence of CIMT (59.22%), for which the independently associated factors are age, albuminuria, and reduced glomerular filtration rate estimated by the CKD-EPI formula, using cystatin C as a serum marker.

Except for the study by Wagenknecht et al. [11], which identified a carotid calcification in 57% of men and 65% of women among 175 African-Americans (85%) with hypertension, in the American state of North Carolina, most researches show a lower prevalence of CIMT and plates in carotid arteries than ours, showing an association only with traditional factors for atherosclerotic disease. Kaul et al. [17] investigated the occurrence of CIMT among 1392 asymptomatic Indians aged over 40 years. They found a prevalence of 41% for this type of injury, whereas Kingue et al. [18], having studied a group of 77 adults in Cameroon, found 25% of the individuals with one or more atheroma plaques in carotid arteries.

Age is an important non-modifiable risk factor for atherosclerosis. Some authors consider atherosclerosis a part of the aging process [19–21]. The isolated influence of agein the beginning of the atherosclerotic process remains uncertain. The accelerated vascular injury by age is commonly considered because of increased oxidative stress, leading to inflammation and endothelial dysfunction; however, no definitive mechanisms have been identified [21]. The tissues of elderly animals show an increased generation of reactive oxygen species (ROS) that lead to altered mitochondrial function, vascular cell damage with changes in vascular remodeling associated with age, and oxidation of lipids, which makes them more atherogenic [21,22].

Similar to this study, several authors have demonstrated the association between age and carotid atherosclerotic disease [23–26]. Gijsberts et al. [10], in a meta-analysis that included 60,211 participants from four ethnic groups (White, Black, Asian, and Hispanic), found that an increase in age was related with the CIMT and the greater risk for cardiovascular events in the Black population. In that study, other traditional, great impact factors for atherosclerosis, such as the level of HDL cholesterol and smoking, showed lower association with the CIMT in black individuals.

The influence of urinary loss of albumin on the prevalence of atherosclerotic lesions in the carotid arteries of the Afro-descendants was also demonstrated. Severe albuminuria is considered a structural renal damage marker [27]. It is associated with an increase in cardiovascular risk and mortality in patients with chronic renal disease [28]. Albuminuria represents the presence of endothelial injury and early atherosclerotic vascular disease, serving as a marker of severity of hypertension and a predictor of cardiovascular events [29]. A study conducted by Garimella et al. [30], prospectively evaluating a sample of 6814 patients from the *Multi-Ethnic Study of Atherosclerosis* (average of 9.8 years of follow-up), concluded that albuminuria was an independent risk factor for the development of peripheral arterial disease, after finding that patients with higher ACR quintile had a significant increased risk of the progression of ankle-brachial index below 0.90 (OR, 1.79; 95% CI, 1.03–3.12) and above 1.40 (OR, 2.76; 95% CI, 1.32–5.77), which is a reflection of atherosclerotic disease.

The *Bogalusa Heart Study* [31], including 1193 participants to evaluate subclinical atherosclerotic changes in young adults, of which 30% were of Black race, found that the presence of albuminuria was significantly associated with thickening of the common carotid artery (*p* values = .005). Moreover, in the multivariate logistic regression model, only the Black race was associated with the presence of albuminuria (OR =1.92, *p* values = .005). This study, which included only African-American subjects, also showed a statistically significant association between CIMT ≥ 0.9 mm and ACR ≥30 mg/g, with a *p* values = .037.

The relationship between albuminuria and calcified atherosclerotic plaque has shown ethnic differences, with lower levels of calcified plaque found in African-American individuals in relation to Caucasians with or without diabetes mellitus [32–34]. *The African* ***-*** *American-diabetes heart study* [35], including 835 Caucasians and 393 African-Americans with diabetes mellitus type 2, concluded that albuminuria is strongly associated with severity of calcified plaque in Caucasians (*p* < .01), but not among African-Americans (*p* = .33), after adjustments for age, sex, glomerular filtration rate, and body mass index. In addition, the calcium metabolism clearly differs between African-Americans and European Americans. Although African-Americans typically resent lesser intake of calcium in the diet than Caucasians, they have a higher bone density, with lower rates of osteoporosis, and skeletal resistance to the effects of the parathyroid hormone [36]. The interaction between these variables might contribute to explain ethnic differences in the rates of cardiovascular events observed in patients with diabetes mellitus type 2.

The findings of this study indicate that the GFR estimated by cystatin C has a strong association with CIMT, unlike the glomerular filtration rate estimated using the serum creatinine. Several studies have pointed out that cystatin C shows a stronger association to creatinine in the occurrence of cardiovascular events and mortality [37,38], because cystatin C is involved in protein catabolism, inhibiting elastolytic proteases, which are increased in degenerative and inflammatory processes, such as atherosclerosis, which may work as a direct marker of the atherogenic process [39].

Nevertheless, the number of studies that attempted to correlate the cystatin C with CIMT is still limited. Their results are conflicting. While two small cross-sectional studies involving middle-aged patients with hypertension reported a significant correlation between cystatin C and CMIT [40, 41], larger cross-sectional studies did not confirm this finding, either in individuals with preserved renal function [42] or middle-aged subjects or elderly patients with discreet to moderate renal dysfunction [43]. A cross-sectional study conducted by Potter et al. [44] analyzed a subset of 173 elderly individuals with stroke who were participating in the *Vitamins to Prevent Stroke* (VITATOPS trial). Its main objective was to evaluate the effect of a decrease in homocysteine by supplementation with complex B vitamins in reducing the incidence of greater vascular events. However, it did not identify a correlation between cystatin C and CIMT.

Monteiro et al. [45] evaluated the outpatients with hypertension without manifestations of cardiovascular disease, who were predominantly middle-aged, had preserved renal function, and were classified as low-risk and average, according to the Framingham score. After conducting a multiple regression analysis, they only found an association of serum cystatin C levels with the creatinine clearance (*r* = 0.491, *p* < .001). However, the serum cystatin C was not correlated with the CIMT and flow-mediated dilation of the brachial artery, corroborating the role of cystatin C as a renal function marker. Nevertheless, it did not show an association with atherosclerosis markers.

Only 5 of the 122 subjects diagnosed with CIMT ≥0.9 mm were using statins. Statins have been shown in primary and secondary cardiovascular prevention trials to reduce the incidence of cardiovascular events [46]. It is not clear whether this is attributable to a reduction in low-density lipoprotein cholesterol (LDL-C) or to other pleiotropic effects of statins [47]. A meta-analysis published for Bedi et al. [48] showed that there was a statistically significant benefit with statin therapy in slowing down the progression of CIMT and the common mean difference between statin therapy arm and placebo arm was 0.040 (CI: 0.052–0.028; *p* values < .001). In our study, the use of statin did not alter the prevalence of carotid intima-media thickness.

Although previous studies indicate diabetes mellitus [17], smoking [49], dyslipidemia [50], and uric acid [18] as risk factors for atherosclerosis, in this study, such variables were not independently associated with the occurrence of CIMT in the group of Afro-descendants after the multivariate regression model.

This study has some limitations. The first is related to its cross-sectional design, which prevents forming conclusions on the impact of the markers evaluated on the occurrence of cardiovascular events. Additionally, since a highly specific group of patients was assessed, the results cannot be automatically extrapolated to the Afro-descendant populations. Conversely, our work shows some positive aspects that deserve to be mentioned. This is the first study to diagnose carotid atherosclerotic disease in the Black population descending from the African slave community who live in the isolated places of Brazil. Furthermore, the association of glomerular filtration rate, estimated by cystatin C (not by the creatinine) and albuminuria as renal function markers in an isolated sample, with carotid thickening must be highlighted. These markers might be used in the assessment of atherosclerotic disease in patients with similar social, demographic, and clinical characteristics.

In conclusion, the occurrence of carotid atherosclerotic lesions measured using CIMT was high in the studied group. Renal disease markers (GFR and albuminuria) and age were independently associated with these lesions. The reduced GFR influenced the prevalence of carotid atherosclerosis only when calculated by the CKD-EPI formula using cystatin C as a serum marker. Therefore, the Afro-descendants belonging to the ethnic minority groups, of advanced age, with albuminuria and a reduced GFR (estimated by the CKD-EPI formula using cystatin C) should be strictly monitored and treated to reduce the risk of cardiovascular events, considering their association with carotid atherosclerotic disease.
